# Methyl 2-[(*Z*)-5-bromo-2-oxoindolin-3-yl­idene]­hydrazinecarbodi­thio­ate

**DOI:** 10.1107/S2414314624007879

**Published:** 2024-08-16

**Authors:** Mohd Abdul Fatah Abdul Manan, David B. Cordes, Aidan P. McKay

**Affiliations:** a Faculty of Applied Sciences, Universiti Teknologi MARA, 40450 Shah Alam, Selangor, Malaysia; b EaStCHEM School of Chemistry, University of St Andrews, St Andrews, Fife KY16 9ST, United Kingdom; University of Aberdeen, United Kingdom

**Keywords:** crystal structure, di­thio­carbazate, 5-bromo­isatin, *Z* configuration, hydrogen bonding, halogen bond

## Abstract

The title compound, C_10_H_8_BrN_3_OS_2_, a brominated di­thio­carbazate imine deriv­ative, was obtained from the condensation reaction of *S*-methyl­dithio­carbazate (SMDTC) and 5-bromo­isatin. The essentially planar mol­ecule exhibits a *Z* configuration, with the di­thio­carbazate and 5-bromo­isatin fragments located on the same sides of the C=N azomethine bond, which allows for the formation of an intra­molecular N—H⋯O_b_ (b = bromo­isatin) hydrogen bond generating an *S*(6) ring motif. In the crystal, adjacent mol­ecules are linked by pairs of N—H⋯O hydrogen bonds, forming dimers characterized by an *R*^2^_2_(8) loop motif. In the extended structure, mol­ecules are linked into a three-dimensional network by C—H⋯S and C—H⋯Br hydrogen bonds, C—Br⋯S halogen bonds and aromatic π–π stacking.

## Abstract 2

The crystal structure of a new *S*-methyl-substituted di­thio­carbazate imine containing the 5-bromo­isatin moiety is described.

## Graphical abstract

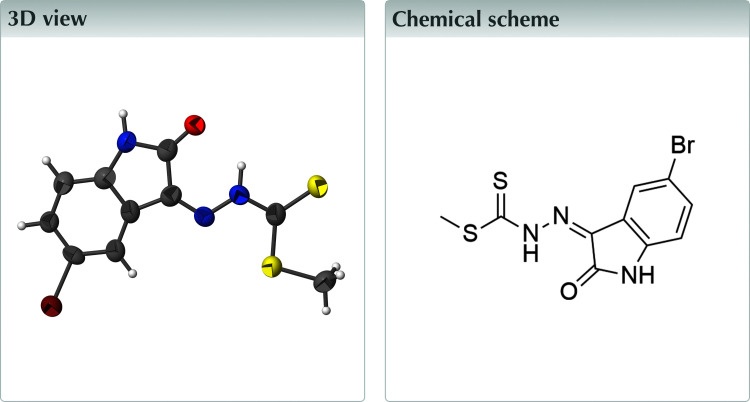

## Structure description

Isatin-derived di­thio­carbazate imines have been reported to exhibit a broad spectrum of physiological properties (Yekke-ghasemi *et al.*, 2020 ▸; Ramilo-Gomes *et al.*, 2021 ▸). In particular, the *S*-methyl-substituted derivatives have received considerable attention in the field of organic transformations for the preparation of carbo­thio­hydrazones, carbo­thio­hydrazides and various aza-heterocyclic compounds such as pyrazoles, 1,2,4-triazoles and 1,3,4-thia­diazo­les (Lin *et al.*, 2013 ▸; Moustafa *et al.*, 2021 ▸; Bekircan *et al.*, 2022 ▸; Malakar *et al.*, 2023 ▸, Geoghegan *et al.*, 2024 ▸). Recent study has revealed that the imine obtained from the condensation reaction of isatin and *S*-methyl­dithio­carbazate can be directly transformed into the spiro-fused 1,3,4-thia­diazole compound in a straightforward synthetic protocol (Moustafa *et al.*, 2021 ▸). This approach opens a new avenue in accessing isatin-based spiro­cycle mol­ecules. We are concerned with developing new di­thio­carbazate imines containing isatin derivatives and continue research work to explore their potential applications (Abdul Manan *et al.*, 2024 ▸). Therefore, as part of our ongoing research and structural studies on such mol­ecules, we now report the synthesis and crystal structure of the title compound.

**Figure 1:**
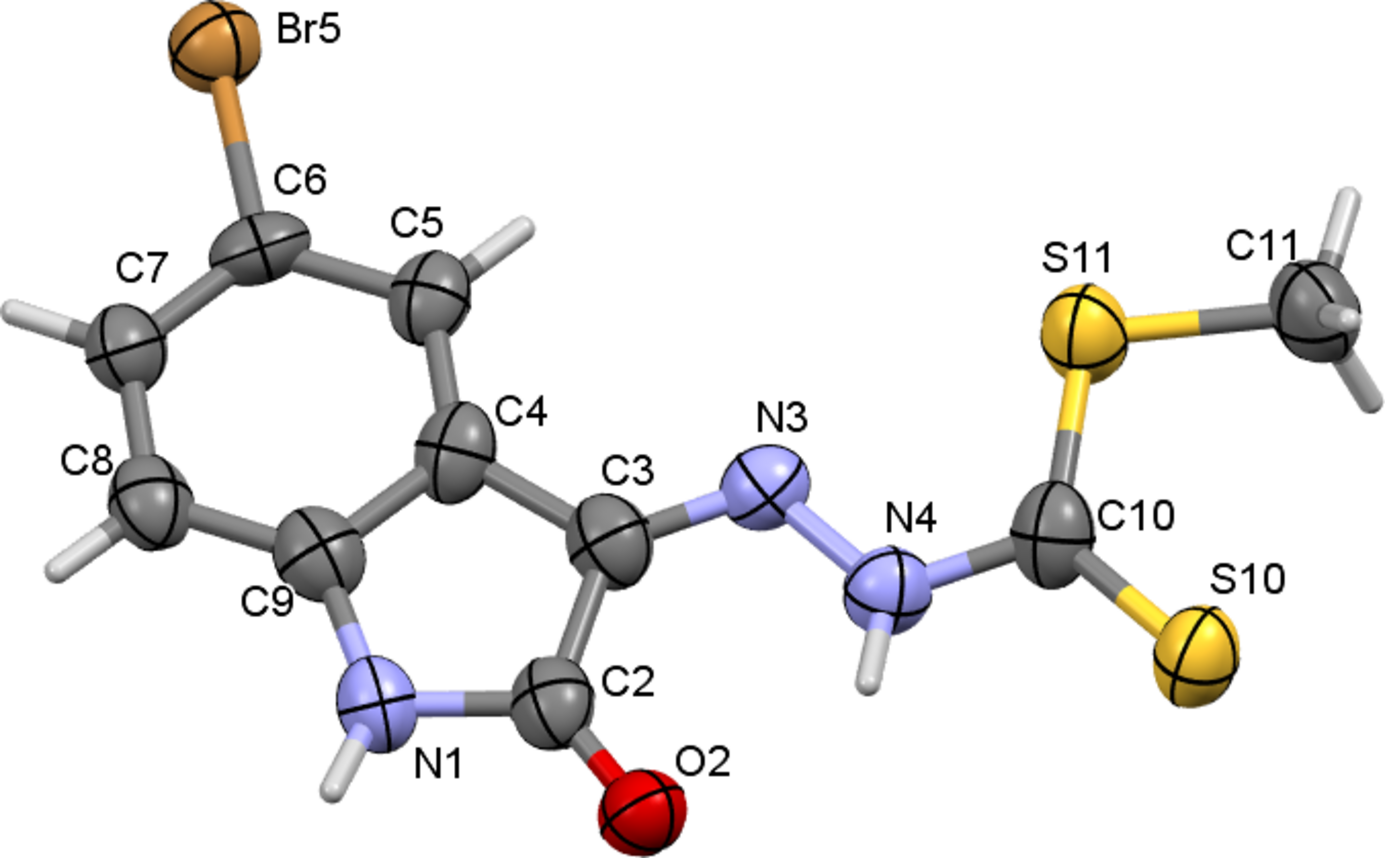
The mol­ecular structure of the title compound, showing displacement ellipsoids drawn at the 50% probability level.

The asymmetric unit of the title compound, C_10_H_8_BrN_3_OS_2_ contains one mol­ecule and crystallizes in the *P*2_1_/*n* monoclinic space group with the *S*-methyl and thione groups being *syn* (Fig. 1 ▸). The non-hydrogen atoms in the mol­ecule are close to planar, indicating electron delocalization within the mol­ecule: the dihedral angle between the di­thio­carbazate group and the 5-bromo­isatin ring is 7.9 (3)°. The imine exists in its thione tautomeric form with the di­thio­carbazate unit adopting a *Z* conformation about the C=N bond [C4—C3=N3—N4 = 177.9 (9)°] with respect to the 5-bromo­isatin moiety, while the S10 thio­keto sulfur atom is positioned *anti* to the N3 azomethine nitro­gen atom [N3—N4—C10—S10 = 173.8 (7)°]. The presence of an N—H⋯O_b_ (b = bromo­isatin) intra­molecular hydrogen helps to consolidate the planar conformatic of the title mol­ecule (Table 1 ▸). Otherwise, the bond lengths and angles are comparable to those reported for methyl 2-(5-chloro-2-oxo-1,2-di­hydro-3*H*-indol-3-yl­idene)hydrazine­carbodi­thio­ate (Abdul Manan *et al.*, 2011 ▸), methyl 2-(1-methyl-2-oxo-1,2-di­hydro-3*H*-indol-3-yl­idene)hydrazine­carbodi­thio­ate (Abdul Manan *et al.*, 2012 ▸) and methyl (*Z*)-2-(5-fluoro-2-oxo-1,2-di­hydro-3*H*-indol-3-yl­idene)hydrazine-1-carbodi­thio­ate (Li *et al.*, 2018 ▸).

**Figure 2:**
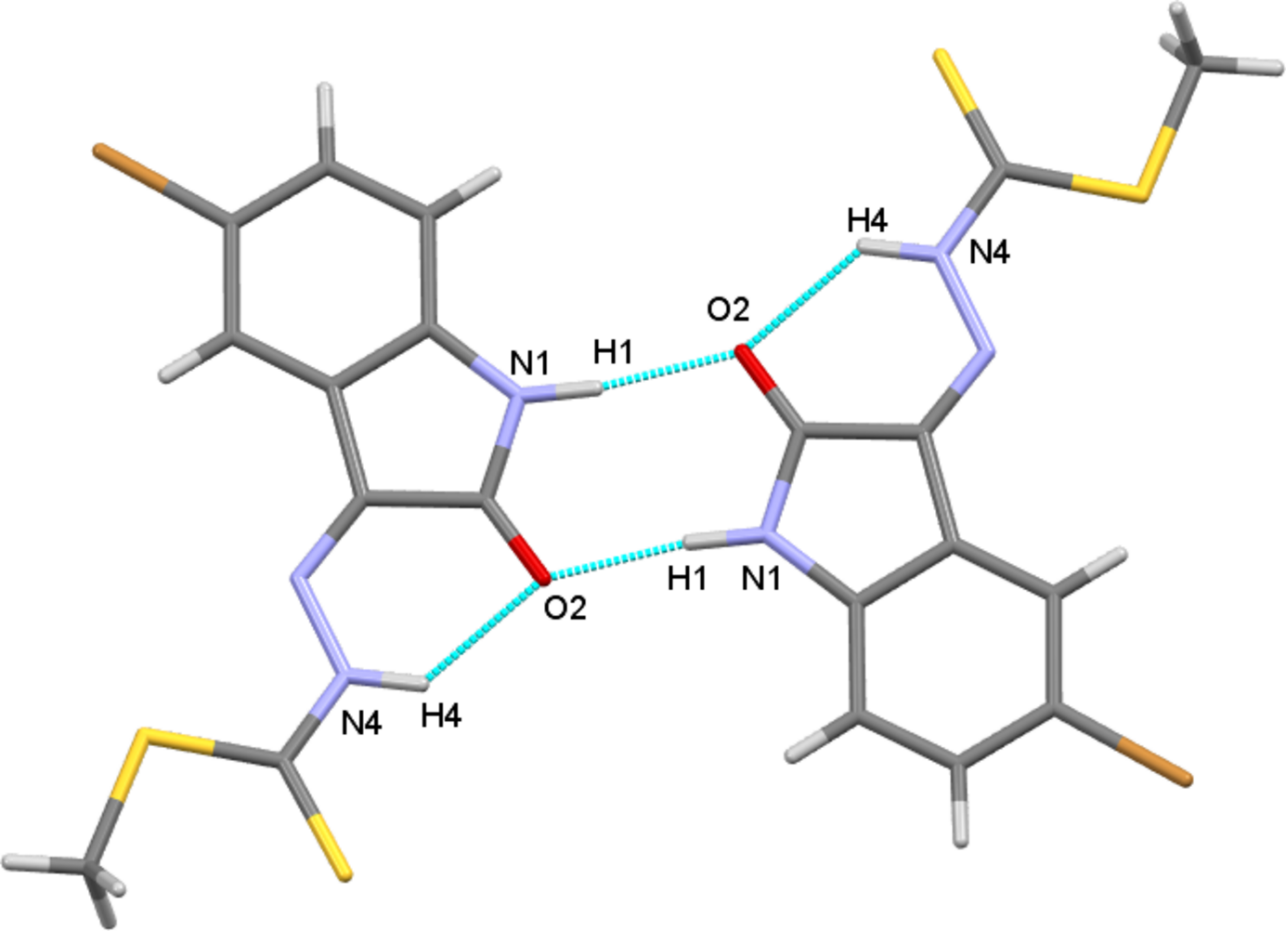
View of the dimers formed by N—H⋯O hydrogen-bonding giving 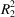
(8) motifs.

**Figure 3:**
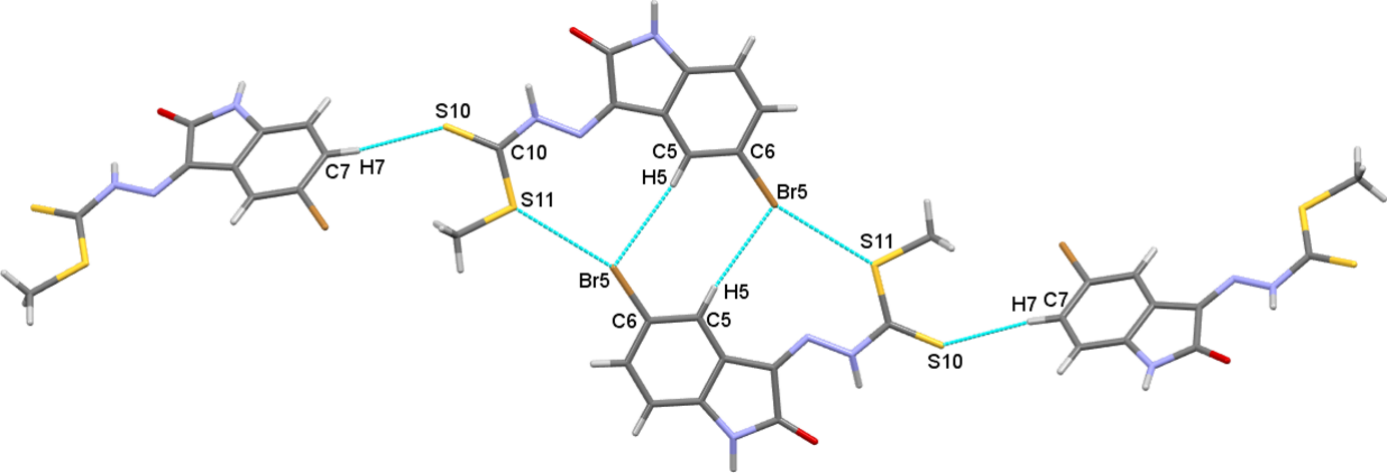
View showing both the weak hydrogen bonds and halogen bonds connecting the hydrogen-bonded dimers in three-dimensions.

In the crystal of the title compound, mol­ecules are linked into dimers through pairwise N1—H1⋯O2 hydrogen bonds in a common 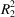
(8) motif (Fig. 2 ▸). These dimers further pack into chains propagating along [2
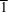
0] through a combination of weak C5—H5⋯Br5 hydrogen bonds and Br5⋯S11 [Br⋯S = 3.521 (3) Å, C—S⋯Br = 135.1 (3)°, C—Br⋯S = 155.2 (3)°] halogen bonds, forming a extended 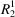
(9)
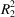
(8)
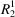
(9) motif. These chains are connected into the third dimension by further weak C7—H7⋯S10 hydrogen bonds (Table 1 ▸, Fig. 3 ▸). These hydrogen bonded arrays have parallel mol­ecules separated such that an equivalent inter­penetrating mol­ecular framework exists, inter­acting with the other *via* aromatic π–π stacking [N1/C2–C4/C9, centroid⋯centroid separation = 3.307 (9) Å].

## Synthesis and crystallization

The di­thio­carbazate precursor, SMDTC was prepared by the literature method (Das & Livingstone, 1976 ▸). The title compound was prepared by adding 5-bromo­isatin (2.26 g, 10.0 mmol, 1.0 eq) dissolved in hot ethanol (20 ml) to a solution of the precursor, SMDTC (1.22 g, 10.0 mmol, 1.0 eq) in hot ethanol (35 ml). The mixture was heated (80°C) with continuous stirring for 15 min and later allowed to stand about 20 min at room temperature until a precipitate was formed, which was then filtered and dried over silica gel, yielding orange crystals on recrystallization from ethanol solution (yield: 2.74 g, 83%). m.p. 259–260°C; ^1^H NMR (400 MHz, *d*_6_-DMSO) δ: (p.p.m.): 2.63 (*s*, 3H), 6.92 (*d*, *J* = 8.28 Hz, 1H), 7.59 (*dd*, *J* = 8.32, 2.0 Hz, 1H), 7.63 (*d*, *J* = 1.92 Hz, 1H), 11.48 (*s*, 1H), 13.90 (*s*, 1H); HRMS *m*/*z* (ESI^+^), found: [*M* + H]^+^ 329.9335, C_10_H_8_N_3_OS_2_^79^Br requires [*M* + H]^+^ 329.9370.

## Refinement

Crystal data, data collection and structure refinement details are summarized in Table 2 ▸. The structure was refined as a two-component twin with component 2 rotated by −176.99° around [0.92 0.02 − 0.38] (reciprocal) or [1.00 0.02 0.00] (direct), and a refined twin fraction of 0.056 (3).

## Supplementary Material

Crystal structure: contains datablock(s) I. DOI: 10.1107/S2414314624007879/hb4481sup1.cif

Structure factors: contains datablock(s) I. DOI: 10.1107/S2414314624007879/hb4481Isup2.hkl

Supporting information file. DOI: 10.1107/S2414314624007879/hb4481Isup3.cml

CCDC reference: 2376721

Additional supporting information:  crystallographic information; 3D view; checkCIF report

## Figures and Tables

**Table 1:** Hydrogen-bond geometry (Å, °)

*D*—H⋯*A*	*D*—H	H⋯*A*	*D*⋯*A*	*D*—H⋯*A*
N4—H4⋯O2	0.98 (2)	2.04 (8)	2.715 (11)	124 (7)
N1—H1⋯O2^i^	0.97 (2)	1.86 (4)	2.801 (11)	163 (10)
C5—H5⋯Br5^ii^	0.95	3.00	3.941 (10)	172
C7—H7⋯S10^iii^	0.95	2.83	3.775 (10)	171

Symmetry codes: (i) 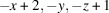; (ii) 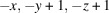; (iii) 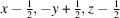.

**Table 2:** Experimental details

Crystal data	
Chemical formula	C_10_H_8_BrN_3_OS_2_
*M*_r_	330.22
Crystal system, space group	Monoclinic, *P*2_1_/*n*
Temperature (K)	125
*a*, *b*, *c* (Å)	6.6331 (3), 7.5726 (3), 24.6985 (10)
β (°)	97.141 (4)
*V* (Å^3^)	1230.98 (9)
*Z*	4
Radiation type	Cu *K*α
μ (mm^−1^)	7.63
Crystal size (mm)	0.11 × 0.01 × 0.01 × 0.40 (radius)

Data collection	
Diffractometer	Rigaku XtaLAB P200K
Absorption correction	Multi-scan (*CrysAlis PRO*; Rigaku OD, 2023 ▸)
*T*_min_, *T*_max_	0.025, 0.114
No. of measured, independent and observed [*I* > 2σ(*I*)] reflections	11989, 2513, 1869
*R*_int_	0.1931
(sin θ/λ)_max_ (Å^−1^)	0.630

Refinement	
*R*[*F*^2^ > 2σ(*F*^2^)], *wR*(*F*^2^), *S*	0.093, 0.293, 1.14
No. of reflections	2513
No. of parameters	164
No. of restraints	2
H-atom treatment	H atoms treated by a mixture of independent and constrained refinement
Δρ_max_, Δρ_min_ (e Å^−3^)	1.37, −1.06

Computer programs: *CrysAlis PRO* (Rigaku OD, 2023 ▸), *SHELXT* (Sheldrick, 2015*a* ▸), *SHELXL2019/3* (Sheldrick, 2015*b* ▸), *Mercury* (Macrae *et al.*, 2020 ▸), *OLEX2* (Dolomanov *et al.*, 2009 ▸) and *publCIF* (Westrip, 2010 ▸).

## full crystallographic data

### Methyl 2-[(Z)-5-bromo-2-oxoindolin-3-ylidene]hydrazinecarbodithioate Crystal data

**Table:**

C_10_H_8_BrN_3_OS_2_	*F*(000) = 656
*M**_r_* = 330.22	*D*_x_ = 1.782 Mg m^−^^3^
Monoclinic, *P*2_1_/*n*	Cu *K*α radiation, λ = 1.54184 Å
*a* = 6.6331 (3) Å	Cell parameters from 3279 reflections
*b* = 7.5726 (3) Å	θ = 3.6–75.3°
*c* = 24.6985 (10) Å	µ = 7.63 mm^−^^1^
β = 97.141 (4)°	*T* = 125 K
*V* = 1230.98 (9) Å^3^	Needle, orange
*Z* = 4	0.11 × 0.01 × 0.01 × 0.40 (radius) mm

### Methyl 2-[(Z)-5-bromo-2-oxoindolin-3-ylidene]hydrazinecarbodithioate Data collection

**Table:**

Rigaku XtaLAB P200K diffractometer	2513 independent reflections
Radiation source: Rotating Anode, Rigaku MM-007HF	1869 reflections with *I* > 2σ(*I*)
Rigaku Osmic Confocal Optical System monochromator	*R*_int_ = 0.193
Detector resolution: 5.8140 pixels mm^-1^	θ_max_ = 76.3°, θ_min_ = 6.1°
shutterless scans	*h* = −7→7
Absorption correction: multi-scan (CrysAlisPr; Rigaku OD, 2023)	*k* = −9→9
*T*_min_ = 0.025, *T*_max_ = 0.114	*l* = −5→30
11959 measured reflections	

### Methyl 2-[(Z)-5-bromo-2-oxoindolin-3-ylidene]hydrazinecarbodithioate Refinement

**Table:**

Refinement on *F*^2^	Primary atom site location: dual
Least-squares matrix: full	Hydrogen site location: mixed
*R*[*F*^2^ > 2σ(*F*^2^)] = 0.093	H atoms treated by a mixture of independent and constrained refinement
*wR*(*F*^2^) = 0.293	*w* = 1/[σ^2^(*F*_o_^2^) + (0.145*P*)^2^ + 4.1265*P*] where *P* = (*F*_o_^2^ + 2*F*_c_^2^)/3
*S* = 1.14	(Δ/σ)_max_ < 0.001
2513 reflections	Δρ_max_ = 1.37 e Å^−^^3^
164 parameters	Δρ_min_ = −1.06 e Å^−^^3^
2 restraints	

### Methyl 2-[(Z)-5-bromo-2-oxoindolin-3-ylidene]hydrazinecarbodithioate Special details

**Table:**

Geometry. All esds (except the esd in the dihedral angle between two l.s. planes) are estimated using the full covariance matrix. The cell esds are taken into account individually in the estimation of esds in distances, angles and torsion angles; correlations between esds in cell parameters are only used when they are defined by crystal symmetry. An approximate (isotropic) treatment of cell esds is used for estimating esds involving l.s. planes.
Refinement. Refined as a 2-component twin with HKLF5 generated by TWINROTMAT running in PLATON. NH hydrogen atoms were identifed from F_map_ and refined with N-H restrained to 0.98 Å.

### Methyl 2-[(Z)-5-bromo-2-oxoindolin-3-ylidene]hydrazinecarbodithioate Fractional atomic coordinates and isotropic or equivalent isotropic displacement parameters (Å^2^)

**Table:**

	*x*	*y*	*z*	*U*_iso_*/*U*_eq_	
Br5	−0.00632 (16)	0.49654 (14)	0.40002 (4)	0.0565 (4)	
S11	0.3366 (4)	0.2379 (4)	0.68815 (10)	0.0606 (7)	
S10	0.7209 (4)	0.0479 (4)	0.73636 (10)	0.0602 (7)	
O2	0.9075 (10)	0.0482 (9)	0.5665 (3)	0.0519 (15)	
N1	0.7784 (13)	0.1254 (11)	0.4779 (3)	0.0497 (17)	
N4	0.6432 (12)	0.1539 (11)	0.6357 (3)	0.0500 (17)	
N3	0.5194 (11)	0.2188 (10)	0.5925 (3)	0.0471 (17)	
C2	0.7769 (15)	0.1156 (13)	0.5335 (4)	0.051 (2)	
C6	0.2344 (13)	0.3758 (12)	0.4224 (4)	0.048 (2)	
C9	0.5992 (16)	0.2052 (13)	0.4534 (4)	0.052 (2)	
C4	0.4781 (16)	0.2581 (12)	0.4928 (4)	0.050 (2)	
C5	0.2935 (15)	0.3440 (12)	0.4784 (4)	0.0483 (19)	
H5	0.210762	0.379717	0.505144	0.058*	
C10	0.5760 (17)	0.1466 (13)	0.6859 (4)	0.054 (2)	
C7	0.3544 (14)	0.3217 (13)	0.3840 (4)	0.051 (2)	
H7	0.309938	0.343966	0.346543	0.061*	
C11	0.3089 (19)	0.2206 (17)	0.7602 (5)	0.065 (3)	
H11A	0.175584	0.266410	0.766485	0.098*	
H11B	0.415852	0.289473	0.781506	0.098*	
H11C	0.320165	0.096497	0.771421	0.098*	
C8	0.5376 (16)	0.2359 (13)	0.3979 (4)	0.053 (2)	
H8	0.618622	0.199134	0.370855	0.064*	
C3	0.5842 (16)	0.2045 (13)	0.5453 (4)	0.052 (2)	
H4	0.753 (10)	0.069 (10)	0.633 (4)	0.04 (2)*	
H1	0.881 (13)	0.046 (12)	0.467 (4)	0.05 (3)*	

### Methyl 2-[(Z)-5-bromo-2-oxoindolin-3-ylidene]hydrazinecarbodithioate Atomic displacement parameters (Å^2^)

**Table:**

	*U*^11^	*U*^22^	*U*^33^	*U*^12^	*U*^13^	*U*^23^
Br5	0.0560 (7)	0.0579 (7)	0.0547 (7)	0.0055 (4)	0.0036 (5)	0.0015 (4)
S11	0.0661 (15)	0.0663 (15)	0.0499 (13)	0.0133 (12)	0.0089 (11)	0.0043 (11)
S10	0.0692 (16)	0.0626 (15)	0.0472 (13)	0.0112 (12)	0.0007 (11)	0.0017 (11)
O2	0.049 (4)	0.055 (4)	0.051 (3)	0.004 (3)	0.002 (3)	−0.001 (3)
N1	0.060 (5)	0.048 (4)	0.043 (4)	0.000 (3)	0.012 (3)	−0.001 (3)
N4	0.052 (4)	0.047 (4)	0.050 (4)	0.007 (3)	0.004 (3)	0.002 (3)
N3	0.049 (4)	0.042 (4)	0.050 (4)	0.002 (3)	0.002 (3)	0.000 (3)
C2	0.058 (5)	0.047 (5)	0.047 (5)	−0.009 (4)	0.008 (4)	−0.006 (4)
C6	0.034 (4)	0.046 (4)	0.061 (5)	0.000 (3)	−0.008 (4)	0.001 (4)
C9	0.060 (6)	0.043 (5)	0.056 (5)	−0.005 (4)	0.013 (4)	−0.001 (4)
C4	0.067 (6)	0.038 (4)	0.043 (4)	−0.003 (4)	0.002 (4)	−0.003 (3)
C5	0.058 (5)	0.042 (4)	0.044 (5)	−0.007 (4)	0.002 (4)	−0.002 (3)
C10	0.074 (6)	0.043 (5)	0.046 (5)	0.004 (4)	0.007 (4)	−0.002 (4)
C7	0.052 (5)	0.055 (5)	0.046 (5)	−0.002 (4)	0.009 (4)	0.006 (4)
C11	0.070 (7)	0.070 (7)	0.058 (6)	0.009 (5)	0.019 (5)	−0.004 (5)
C8	0.060 (6)	0.052 (5)	0.049 (5)	−0.011 (4)	0.013 (4)	0.000 (4)
C3	0.059 (5)	0.046 (5)	0.052 (5)	−0.001 (4)	0.012 (4)	−0.004 (4)

### Methyl 2-[(Z)-5-bromo-2-oxoindolin-3-ylidene]hydrazinecarbodithioate Geometric parameters (Å, º)

**Table:**

Br5—C6	1.863 (8)	C6—C5	1.410 (13)
S11—C10	1.739 (11)	C6—C7	1.375 (14)
S11—C11	1.816 (11)	C9—C4	1.395 (14)
S10—C10	1.654 (10)	C9—C8	1.399 (14)
O2—C2	1.224 (12)	C4—C5	1.394 (14)
N1—C2	1.378 (12)	C4—C3	1.455 (13)
N1—C9	1.402 (13)	C5—H5	0.9500
N1—H1	0.97 (2)	C7—H7	0.9500
N4—N3	1.355 (11)	C7—C8	1.383 (15)
N4—C10	1.371 (13)	C11—H11A	0.9800
N4—H4	0.98 (2)	C11—H11B	0.9800
N3—C3	1.294 (12)	C11—H11C	0.9800
C2—C3	1.505 (14)	C8—H8	0.9500
			
C10—S11—C11	101.9 (5)	C4—C5—C6	117.3 (9)
C2—N1—C9	110.1 (8)	C4—C5—H5	121.3
C2—N1—H1	110 (7)	S10—C10—S11	127.1 (6)
C9—N1—H1	137 (7)	N4—C10—S11	114.4 (7)
N3—N4—C10	119.6 (8)	N4—C10—S10	118.4 (8)
N3—N4—H4	124 (5)	C6—C7—H7	118.9
C10—N4—H4	111 (5)	C6—C7—C8	122.3 (9)
C3—N3—N4	116.2 (8)	C8—C7—H7	118.9
O2—C2—N1	126.5 (9)	S11—C11—H11A	109.5
O2—C2—C3	127.3 (9)	S11—C11—H11B	109.5
N1—C2—C3	106.3 (8)	S11—C11—H11C	109.5
C5—C6—Br5	119.9 (7)	H11A—C11—H11B	109.5
C7—C6—Br5	119.3 (7)	H11A—C11—H11C	109.5
C7—C6—C5	120.8 (8)	H11B—C11—H11C	109.5
C4—C9—N1	110.7 (9)	C9—C8—H8	121.2
C4—C9—C8	120.8 (10)	C7—C8—C9	117.5 (9)
C8—C9—N1	128.5 (9)	C7—C8—H8	121.2
C9—C4—C3	106.6 (9)	N3—C3—C2	126.6 (9)
C5—C4—C9	121.3 (9)	N3—C3—C4	126.9 (9)
C5—C4—C3	132.1 (9)	C4—C3—C2	106.3 (8)
C6—C5—H5	121.3		
			
Br5—C6—C5—C4	−178.0 (7)	C9—N1—C2—O2	177.5 (9)
Br5—C6—C7—C8	178.1 (8)	C9—N1—C2—C3	−2.8 (10)
O2—C2—C3—N3	−3.0 (17)	C9—C4—C5—C6	−0.2 (14)
O2—C2—C3—C4	−178.5 (9)	C9—C4—C3—N3	−175.6 (10)
N1—C2—C3—N3	177.3 (9)	C9—C4—C3—C2	−0.2 (10)
N1—C2—C3—C4	1.8 (10)	C4—C9—C8—C7	0.6 (14)
N1—C9—C4—C5	178.7 (8)	C5—C6—C7—C8	−0.6 (15)
N1—C9—C4—C3	−1.6 (11)	C5—C4—C3—N3	4.0 (17)
N1—C9—C8—C7	−178.4 (9)	C5—C4—C3—C2	179.5 (10)
N4—N3—C3—C2	3.3 (14)	C10—N4—N3—C3	−172.9 (9)
N4—N3—C3—C4	177.9 (9)	C7—C6—C5—C4	0.7 (13)
N3—N4—C10—S11	−3.9 (12)	C11—S11—C10—S10	6.9 (9)
N3—N4—C10—S10	173.8 (7)	C11—S11—C10—N4	−175.7 (8)
C2—N1—C9—C4	2.9 (11)	C8—C9—C4—C5	−0.5 (14)
C2—N1—C9—C8	−178.0 (10)	C8—C9—C4—C3	179.2 (9)
C6—C7—C8—C9	−0.1 (15)	C3—C4—C5—C6	−179.8 (10)

### Methyl 2-[(Z)-5-bromo-2-oxoindolin-3-ylidene]hydrazinecarbodithioate Hydrogen-bond geometry (Å, º)

**Table:**

*D*—H···*A*	*D*—H	H···*A*	*D*···*A*	*D*—H···*A*
N4—H4···O2	0.98 (2)	2.04 (8)	2.715 (11)	124 (7)
N1—H1···O2^i^	0.97 (2)	1.86 (4)	2.801 (11)	163 (10)
C5—H5···Br5^ii^	0.95	3.00	3.941 (10)	172
C7—H7···S10^iii^	0.95	2.83	3.775 (10)	171

Symmetry codes: (i) −*x*+2, −*y*, −*z*+1; (ii) −*x*, −*y*+1, −*z*+1; (iii) *x*−1/2, −*y*+1/2, *z*−1/2.
